# Supplementary material for: Genotypic distribution and molecular epidemiology of HPV in women in the UAE using PNA-based RT PCR
Source: PLoS One. 2026 Mar 31;21(3):e0346052. doi: 10.1371/journal.pone.0346052 (PMC13037986; doi:10.1371/journal.pone.0346052)
Supplement: S1 File — Describe the comparability of assessment methods if there is more than one group. (DOCX) [file pone.0346052.s001.docx]

**Data sources/ measurement**

8* For each variable of interest, give sources of data and details of methods of assessment (measurement). Describe comparability of assessment methods if there is more than one group

A total of 04 different age groups were concluded in this study population. Among them, 77 (33.62%) patients aged between 20-30 years, 96 (41.92%) patients fall in the 31-40 age group, the 41 – 50 years old age group included 48 (20.96%) patients, and 08 (3.5%) patients were the age group of above 51 years-old and these details are given in the **Table 1**, **2 & 3**. All the patients were grouped into two ethnicities: Arab and non-Arab. A total of 119 patients (51.96%) were Arab, among them 39 (17.1%) tested positive for HPV infection and 80 (34.93%) were negative. 110 cases (48.04%) were grouped under non-Arab; in that, 57 (24.9%) tested positive and 53 (23.2%) were negative to HPV infection.

Detailed descriptive data of the patients’ age group, ethnicity, HPV positivity, single HPV genotype detection with low risk, high risk genotypes and multiple genotypes with low risk and high-risk genotypes are presented in **Table 2**.

**Table 1.** Detection of HPV infection in different cytology grading in different age group of study population. **(Page No.9)**

**Table 2.** Number of single, multiple low and high-risk HPV genotypes in different cytology samples and different age group of the study population. **(Page No. 11)**

**Table 3.** Frequency of single, multiple low and high-risk HPV genotypes with different age group of the study population a) Age group 20–30; b) 31–40; c) 41–50; d) above 51. **(page No. 16)**.

All the above-mentioned tables (Table 1, 2 & 3) are given in the manuscript.
