# Supplementary material for: Genotypic distribution and molecular epidemiology of HPV in women in the UAE using PNA-based RT PCR
Source: PLoS One. 2026 Mar 31;21(3):e0346052. doi: 10.1371/journal.pone.0346052 (PMC13037986; doi:10.1371/journal.pone.0346052)
Supplement: S2 File — (DOCX) [file pone.0346052.s002.docx]

**Participants**

**13* (a) Report numbers of individuals at each stage of study—eg. numbers potentially eligible, examined for eligibility, confirmed eligible, included in the study, completing follow-up, and analysed**

**Number of individuals at each stage of study**

1. Number of potentially eligible individuals: Women aged 20–55 years attending the gynaecology OPD at Thumbay University Hospital (TUH) and other UAE clinics and hospitals of UAE.
2. Samples received for examination: In total, 229 Liquid-based cervical cytology samples were received from the Thumbay hospitals and other clinics and hospitals of the UAE to the Department of Pathology and Microbiology at Thumbay Laboratory, TUH, Ajman, UAE, to diagnose cervical abnormalities and detect the HPV positivity and their genotypes using the real-time PCR method.
3. Examined for eligibility: All 229 samples assessed for suitability and adequacy of Liquid-based cervical cytology samples for Cytodiagnostic examination and molecular genotyping of HPV by the real-time PCR analysis.
4. Confirmed eligible and included in study: The study populations who visited the hospitals for maiden screening purposes due to the following symptoms, like watery discharge, blood, pelvic pain, post coital bleeding, and pain during sexual intercourse. Those study populations’ liquid-based cervical cytology samples are included for this study, and the patients with a known history of HPV infection or cervical cancer, as well as those previously diagnosed with HPV and undergoing routine follow-up examinations, were excluded from this study. After confirming the eligibility of the samples, they were immediately processed for cytological examination and detection of HPV positivity and their genotypes as per the laboratory protocol.
5. Completed laboratory processing (cytology + HPV testing): All eligible samples (*n=229*) were successfully processed for cytological evaluation, and HPV detection and molecular genotyping were also done.
6. Analysed for HPV positivity and genotype identification: All the 229 processed samples were included in the final data analysis; results documented as cytological detection as
7. LSIL – Low-grade intraepithelial lesion
8. ASCUS – Atypical glandular cell
9. ASC-H – Atypical Squamous cell cannot rule out high-grade squamous intraepithelial cells
10. NILM – Negative for intraepithelial malignancy

HPV positive and negative statuses were detected in the LBC samples, along with high-risk (HR-HPV)and low-risk HPV (LR-HPV) genotypes were also detected using the real-time PCR method.

vii) Follow-Up and Analysis: The sample and patient’s follow-up are not applicable. Because it is a cross-sectional, laboratory-data-based study.

Data Analysis: HPV positivity and genotype results were documented for all 229 samples. Cytological findings were correlated with HPV detection results.
