# Supplementary material for: Genotypic distribution and molecular epidemiology of HPV in women in the UAE using PNA-based RT PCR
Source: PLoS One. 2026 Mar 31;21(3):e0346052. doi: 10.1371/journal.pone.0346052 (PMC13037986; doi:10.1371/journal.pone.0346052)
Supplement: S3 file — (DOCX) [file pone.0346052.s003.docx]

**Liquid Based cervical cytology samples (*n=229*) =(((229(n=*104*)**

**S3. Flow diagram of the study - *ASUCUS** (Atypical squamous cells of undetermined significance), **LSIL** (low-grade squamous intraepithelial lesion), **HSIL** (High-grade squamous intraepithelial lesion), **ASC-H** (Atypical squamous cells, cannot rule out high grade squamous intraepithelial cell), **NILM** (Negative for Intraepithelial Lesion or Malignancy).

HPV18,31,35

HPV16,35,68

HPV16,35,53

HPV16,51,58

HPV59,73

HPV18,31,53,58,68

HPV33,53

HPV56,66

HPV16,31,73

HPV16

HPV31

HPV51

HPV53

HPV58

HPV59

Multiple HR HPV *(n=9)*

HPV42,44

HPV43,61,81

HPV16

HPV53 *(n=2)*

HPV66 *(n=2)*

HPV43,68,81, HPV6,35

HPV44,62, HPV6,66

HPV51,81 *(n=3),* HPV 6,16,18,66

HPV42, 53, HPV 11,31,52

HPV34, 53,82

HPV45,51,82

HPV31,39,51

HPV33,53 *(n=3)*

HPV52,58

HPV56,66

HPV16,31

HPV31,68

HPV16,31,73

HPV16,53

HPV 16,66

HPV 11,59

HPV11,16,43,44,53,59

HPV61,82

HPV6,45,59

HPV 11,35,42,52,68

HPV 43,53,58,61,66

HPV 11,26,31,3543,45,56,66

HPV 56,66

HPV 39,56,66,68

HPV 58,73

HPV 34,35

HPV44,45,52 HPV35,45,54,68

HPV31,61 HPV34,53,82

HPV53,61 HPV6,66

HPV16,18,31,35,43,53,61,68,82

HPV 68

Single HR HPV *(n=1)*

HPV 51,81

Multiple LR & HR HPV *(n=1)*

HPV6 (*n=2),* HPV11, HPV42, HPV43*(n=2),* HPV61*(n=3),* HPV72

HPV84, HPV70

HPV6 *(n=4)*

**HPV+ve *(n=2)***

**HPV+ve *(n=1)***

**HPV-ve *(n=2****)*

**HPV+ve (n=18*)***

**HPV+ve *(n=26)***

**HPV-ve *(n=13)***

**HPV-ve *(n=118)***

**ASC-H *(n=1)***

**AGC *(n=2)***

**LSIL *(n=20)***

**ASCUS *(n=39)***

**Abnormal cervical cytology (n=*62*)**

**NILM (n=*167*)**

Multiple HR HPV *(n=12*)

Multiple LR & HR HPV *(n=11)*

Single LR

HPV *(n=10)*

HPV 16 *(n=2),* HPV18*(n=4),* HPV35, HPV45*(n=4)*, HPV53, HPV59, HPV68*(n=3),* HPV70*(n=2),* HPV73

Single HR HPV *(n=19)*

HPV 31

Single HR HPV *(n=1)*

Multiple LR & HR HPV *(n=7)*

**HPV+ve *(n=49)***

Multiple LR & HR HPV *(n=7)*

Multiple HR HPV *(n=4)*

Mixed LR HPV *(n=2*)

Single HR

HPV *(n=5)*

Single HR HPV *(n=6)*

Papanicolaou stained thin prep smeared slides (n=104)

Single LR

HPV *(n=4)*
