# Supplementary material for: Genotypic distribution and molecular epidemiology of HPV in women in the UAE using PNA-based RT PCR
Source: PLoS One. 2026 Mar 31;21(3):e0346052. doi: 10.1371/journal.pone.0346052 (PMC13037986; doi:10.1371/journal.pone.0346052)
Supplement: S4 File — (DOCX) [file pone.0346052.s004.docx]

**Outcome data**

**15* Cross-sectional study—Report numbers of outcome events or summary measures**

A total of 229 liquid based cervical smears samples were processed for the routine cytopathological examination to identify and differentiate the morphological changes of the PAP smear samples. Based on Bethesda system 2014, the samples were graded as ASCUS (n=39; 17.03%), LSIL (n=20; 8.73%;), AGC (n=2; 0.87%) & ASC-H (n=1; 0.43%) and negative for intraepithelial malignancy (NILM) (n=167; 72.92%) and their details are presented in **Table 1**, and **Fig 3**. Out of 229 PAP smear samples, 96 were positive and 133 were negative to HPV infection and it is mentioned in **Table 1** & **Fig 3**.

In total, 191 HPV genotype were encountered in all the 96 positive samples (47 abnormal cytology & 45 NILM). Among them, 116 genotypes were identified in 47 abnormal cytology samples (ASCUS=26; LSIL=18; AGC=2; ASC-H=1) and 75 genotypes were detected in 49 NILM samples. In the abnormal cytology samples, 61 HPV genotypes were identified from the ASCUS samples. Among them, 39 and 22 genotypes were from the high and low-risk groups respectively. HRHPV53 was detected in 7 different ASCUS samples, HR16 in 6 and 31 & 35 were in 5 different ASCUS. HR35, 68, 18, 58 were detected in 5, 4 and 3 different ASCUS respectively. Similarly, LR6 in 5 &LR61 in 3 ASCUS respectively. In the 18 LSIL samples, a total of 51 HPV genotypes were detected. Among them, 34 and 17 genotypes were from the high and low-risk groups. The genotype HR66 was encountered in 6 LSIL samples and HR53, 59, 56 in 4 and HR35 were in 3 different LSIL samples respectively LR16 was detected only in 2 LSILs. Similarly, LR11, 43 were detected in 4 and LR 61 was in 3 different LSILs. There were 3 different genotypes HR51, 31 & LR81 found in 2 AGC cases, and HR68 was found in the ASC-H cytology. In the NILM cytology, the genotype HR16 was detected in 6 samples and HR18, 68, 53 & 31 each were detected in 5 different NILM samples. LR6 was encountered in 5 different NILM and LR81 & 61 in 4, and LR43 in 3 were identified in NILMs respectively. The number of single and multiple, low and high-risk genotypes detected in different cytology samples is given in **Table 2** and **Fig 4**, and the frequency of H&LR genotypes in the HPV positive samples is presented in **Fig 5**.

A total of four different age groups were included in this study population. Among them, 42 (43.75%) patients were aged between 20-30 years, 40 (41.66%) patients fell in the 31-40 age group, 12 (12.5%) patients were in the 41-50 age group, and 2 (2.08%) were in the >50-year-old age group. These details are provided in the [**Table 1**](file:///C:\Users\nazeerullah5433\Downloads\Table%201.%20Detection%20of%20HPV%20infection%20in%20different%20cytology%20grading%20in%20different%20age%20group%20of%20study%20population). All the study population was divided into two ethnicities: Arab and non-Arab. A total of 119 patients (51.96%) were Arab, among whom 39 (17.07%) tested positive for HPV infection and 80 (34.93%) were negative. 110 cases (48.04%) were classified as non-Arab; among them, 57 (24.89%) tested positive and 53 (23.15%) were negative for HPV infection. Detailed descriptive data of the patients’ age group, ethnicity, HPV positivity, and single or multiple HPV genotype detection with low and high-risk genotypes are presented in the [**Table 2**](file:///C:\Users\nazeerullah5433\Downloads\Table%202.%20Number%20of%20single,%20multiple%20low%20and%20high-risk%20HPV%20genotypes%20in)**.**

In total, 80 (41.7%) HPV genotypes were detected in the 20-30 years old age group. Among them, HPV16 was detected in 9 (11.25%) different samples of ASCUS, NILM (4; 5% each) and LSIL (1;1.25%). HPV68 and 53 were identified in 7 (8.75%) of all abnormal cytological samples. HR45 was detected in 5 (6.25%) samples of NILM (4;5%) and ASCUS (1; 1.25%) while other genotypes 18, 66, 35 and 51 were detected in 4 samples of ASCUS, LSIL and NILM, respectively.

In the 30-39-years old study group, 88 (45.83%) genotypes were observed LR6 (10;11.5%), HR31(8; 4.16%), 53 (7; 3.6%), 35 and 66 (6; 6.9% each) were the common genotypes in this age group followed by HR18, 45, 51, 52 and LR61, 81and 42 (4; 4.6% each) in different samples.

In the age group of 41-50 years old, 21 (10.9%) genotypes were encountered. Among them HR16, 31, 73 and 53 (2;11.76% each) were identified most frequently in 2 samples while other genotypes were not as frequently identified. Similarly, a total of 3 (1.56%) different genotypes (HR66, LR6 and 70) were detected in the study population of >50-years old. These details are provided in the **[Table 2](C:\\Users\\nazeerullah5433\\Downloads\\Table 2. Number of single, multiple low and high-risk HPV genotypes in)** and [**Fig 5**](file:///C:\Users\nazeerullah5433\Downloads\Fig%205.%20%20Number%20of%20single%20and%20multiple,%20low%20and%20high-risk%20genotypes%20detected).

In the age group of 20-30-years old of Arab ethnicity, 37 HPV genotypes were detected. Among them HR16 and 53 were detected in 7 (18.9%) and 5 (13.5%) samples respectively. In the non-Arab study group, 43 genotypes were observed, including HR68 and 51 in 6 (13.9%) and 4 (9.3%) samples, respectively. Additionally, HR18, 58 and LR61 were observed 3 (8.1%) samples.

Similarly, a total of 32 genotypes were identified in the Arab study cohort of the age group of 31-40-years old. HR53 and 31 were detected in 6 (18.75%) and 4 (12.5%) samples, respectively, and LR6 was also observed in 5 (15.6%) samples in the study group.

In the non-Arab study cohort of the same age group, 56 genotypes were observed. Among them, HR66, 51, 45, 31 and 52 were detected in 6 (10.7%), 4 (7.1%) and 3 (5.4%) samples respectively. Additionally, LR6 and 81 were also observed in 5 (8.92%) and 4 (7.14%) samples respectively.

In the age group of 41-50-year-old Arab study group, a total of 7 genotypes (HR66, 59, 31, 68 & LR11,6) were identified. Genotype 66 was detected in 2 samples while the rest were encountered in 1 sample each. In the non-Arab study group, 15 genotypes were detected. Genotypes 16,73 & 53 were observed in 2 samples while the rest were detected in only one sample. Similarly, in the age group of >50 years old, 3 genotypes were detected. HR56 & LR6 were detected in the Arab study group, and LR70 was detected in the non-Arab group. These details are provided in [**Fig 6**](file:///C:\Users\nazeerullah5433\Downloads\Fig%206.%20Frequency%20of%20high%20and%20low%20risk%20genotypes%20in%20HPV%20positive%20samples).

Among the 96 HPV positive cytology samples, 46 (47.91%) samples were infected by single HPV genotypes, of which 30 (31.25%) were infected by only high-risk genotypes whereas low-risk single genotypes were detected in 16 (16.66%) samples. Multiple genotypes were detected in 50 (52.08%) samples; among them, 23 (23.96%) were infected with multiple high-risk genotypes, 2 (2.08%) with multiple low-risk genotypes and 25 (26.04%) samples were infected by mixed genotypes of both low and high-risk types. These details are given in the [**Table 2**](file:///C:\Users\nazeerullah5433\Downloads\Table%202.%20Number%20of%20single,%20multiple%20low%20and%20high-risk%20HPV%20genotypes%20in)**.** The frequency of single, multiple high and low-risk HPV genotypes with different age groups of the study population are provided in [**Table 3**](file:///C:\Users\nazeerullah5433\Downloads\Table%203.%20Frequency%20of%20single,%20multiple%20low%20and%20high-risk%20HPV%20genotypes%20with%20different) and [**Fig 7**](file:///C:\Users\nazeerullah5433\Downloads\Fig%207.%20Frequency%20of%20single,%20multiple%20low%20and%20high). The details of women with different nationality with HPV positivity rate and number of single, mixed genotypes of HPV in normal and abnormal cytological samples is given in **Fig** [**8**](file:///C:\Users\nazeerullah5433\Downloads\Fig%208.%20Nested%20pie%20chart%20of%20women%20with) & [**9**](file:///C:\Users\nazeerullah5433\Downloads\Fig%209.%20Number%20of%20single). The prevalence of high and low-risk genotypes HR53 (8.33%), HR16 (7.29%), HR31, 68, 66 & LR6 (6.25%), HR35,45,51 (4.7%) and HR18 and LR43&61 (4.17%) were moderately high in this finding.

***ASCUS** - Atypical squamous cells of undetermined significance, **LSIL** - Low grade squamous intraepithelial lesion, **ASC-H** - Atypical squamous cells cannot rule out high grade squamous intraepithelial cells. **AGC**- Atypical glandular cell, **NILM** – Negative for intraepithelial malignancy.

* **HR-HPV** – High-risk Human Papillomavirus, **LR-HPV** – Low-risk Human Papillomavirus.
