# Supplementary material for: Genotypic distribution and molecular epidemiology of HPV in women in the UAE using PNA-based RT PCR
Source: PLoS One. 2026 Mar 31;21(3):e0346052. doi: 10.1371/journal.pone.0346052 (PMC13037986; doi:10.1371/journal.pone.0346052)
Supplement: S5 File — (DOCX) [file pone.0346052.s005.docx]

**Interpretation**

**20. Cautious overall interpretation of results considering objectives, limitations, multiplicity of analyses, results from similar studies, and other relevant evidence**

The present study aimed to determine the genotypic distribution and molecular epidemiology of Human papillomaviruses in PAP smear samples of women in the UAE using peptide nucleic acid-based fluorescence melting curve analysis method. Out of 229 PAP smear samples, 27.1% of the samples showed abnormal cytology (ASCUS - 17.03%, LSIL - 8.73%, AGC- 0.87% & ASC-H - 0.44%) that is precancerous type and 72.92% appeared normal cytology.

The rate of abnormal cervical cytology detection in the PAP smear sample has increased slightly to 6% compared with a recent study conducted by Odeh et al in 2023 [13]. However, the rate of abnormal cervical cytology identification in the PAP smear samples has increased nearly by 60% in the UAE compared with previous studies conducted out by Ortashi O and Abdalla D in 2019, by Fakhreldin M and Elmasry K and Al-Zaabi et al. in 2015 [32-34]. Similarly, Yuwadee et al stated that 33.8% of the PAP smear samples from women in Thailand showed abnormal cytology (HSIL, ASCUS, LSIL) and 63.2% were NILM [35]. In the present study, 75.8% of the abnormal cytology in the PAP smears tested positive for HPV infection, while 29.3% of NILM samples were positive for HPV infection. Prevalence of HR-HPV genotypes was 49.7%, with a single genotype prevalence of 16.8% and a mixed HR genotype occurrence of 33.5% in the liquid cytology samples. This was higher compared to the prevalence of LR HPV genotypes, which was 26.7%, with a single LR genotype prevalence of 7.33% and a multiple LR occurrence of 19.37%. The same prevalence of HR HPV was observed by Odeh et al, but the LR prevalence rate was higher (49.6%) than the current study and similarly, the study from Iran in 2021 observed a similar pattern of prevalence rate of HR (43%) and LR (33%) genotypes [13, 36].

Higher HPV positivity rate (17.53%) was observed among the age group of 31-40 years in the study population which is closer to a study conducted in Saudi Arabia & in Bahrain where the HPV positivity was higher in the same age group [37, 38]. However, in contrast to our study, Al-Shammari et al & Yu YQ et al mentioned that the higher HPV positivity was noted in the age group of 40-50 years old of the female study population [23, 26].

The present study found variations in HPV genotype distribution among Arab and non-Arab study population. Among the Arab study cohort, the most encountered high-risk (HR) genotype was 53, followed by genotypes 16, 31, 66, 68 and 45 respectively. Similarly in the non-Arab cohort, HR68 was the most frequently identified genotype, followed by 66, 45, 16, 53 and 18. In the case of low-risk (LR) genotypes among the Arab study group, LR6 was the most detectable genotype, followed by 61, 43 and 11 respectively. In the non-Arab group, LR61, 6, 43 and 11 were commonly detected. However, in contrast to the present study, Odeh et al mentioned that the most commonly detectable HR genotype was 45 and 16 in both Arab and non-Arab cohorts. In LR genotype cases, LR6, 11 and 67 were frequently detected genotypes in the Arab cohort, while LR6 and 62/81 were the commonest genotypes in the non-Arab group [13]. The genotype HR18 was detected in both cohorts, but its positivity rate was slightly higher (2.62%) in the non-Arab study cohort than in the Arab (1.57%) group. Similarly, a low positivity rate of HPV18 was detected in the Western Iranian study group [37]. El-Wakil et al reported that the prevalence of HR16 was nearly 89.3% and HR18 was 3.6% among the study population from Egypt [40]. Additionally, HR16 prevalent in the Mainland and Central parts of China, while HR52 was prevalent in the Southern and Eastern part of China [26]. In cases of mixed co-infection of HPV, LR genotypes (11, 43, 81, 61 and 6) were more frequently encountered than HR genotypes (53, 66 and 35). 12.5% of PAP smear samples were infected by ≥ 3 genotypes, with the non-Arab cohort showing a higher (9.4%) genotype combination than the Arab cohort. The maximum number of genotypes (HR16, 18, 31, 68, 35, 53, 82 and LR 43, 61) were detected in a patient sample. In case of mixed co-infection of HPV (LR & HR) in PAP smear samples, HR genotypes were more predominant (26.2%) than LR genotypes (16.8%).

**Conclusion**

The current findings confirm that the UAE has a lower HPV prevalence (42%) compared to a recent study (60.6%) conducted in 2023. According to the study, HRHPV 53, 16, 31, 68, 66, 35 and 45 and LR-HPV 6, 61, 11,44 and 81 were the most common HPV infections in women aged 20 to 55 years old, with a much lower prevalence of HR-HPV18, 51 and 59 and LR HPV 43. A moderate increase in the incidence of HR HPV 53, 16, 31, 68, 66 and LR HPV 6, 61 and 11 was detected. Co-infection with multiple low and high-risk genotypes was present in 26.2% cases, with HPV 53 being the most common genotype followed by HPV 35, 66, 11, 43, 81, 61 and 6. Based on molecular genotyping, 21.4% of normal epithelia tested positive for HPV infections. It is evident that symptomatic women, even with normal epithelia have been infected with different low and high-risk genotypes. The non-Arab study cohort showed a higher HPV positive rate than the Arab cohort which could be due to a multinational floating population with an unknown HPV screening and vaccination status. Therefore, the present study highlights the importance of molecular genotyping to emphasize HPV screening triage. A large population-based study across the UAE is needed to determine the most prevalent genotypes and develop new vaccine strategies to reduce the burden of cervical cancer.

**Limitations of the study**

As of all other studies this study also reported few limitations as follow. The study samples received from various hospitals, clinics and Thumbay hospitals located in and around the northern and other part of the emirates of UAE (Dubai, Sharjah, Ajman, Umm-Al-Quwain, and Fujairah, ) and not received the samples from other regions such as Abu Dhabi & Al-Ain.

The details of the HPV vaccination status and other comorbidity conditions of the study population were unclear.
