# Supplementary material for: Genotypic distribution and molecular epidemiology of HPV in women in the UAE using PNA-based RT PCR
Source: PLoS One. 2026 Mar 31;21(3):e0346052. doi: 10.1371/journal.pone.0346052 (PMC13037986; doi:10.1371/journal.pone.0346052)
Supplement: S1 Checklist — (DOCX) [file pone.0346052.s006.docx]

STROBE Statement—checklist of items that should be included in reports of observational studies.

|  | Item No. | Recommendation | Page  No. | Relevant text from manuscript | |
| --- | --- | --- | --- | --- | --- |
| **Title and abstract** | 1 | (*a*) Indicate the study’s design with a commonly used term in the title or the abstract | **5** | Yes, the study design was given in the Methodology section – **Study Design & Study population** of the manuscript.  “A cross - sectional retrospective study was conducted on the liquid-based cervical cytology samples received from various hospitals of UAE to the Department of Pathology at Thumbay laboratory, Thumbay University Hospital (TUH), Ajman, UAE during the period of January 10, 2024 to January 10, 2025, to diagnose the cervical abnormalities and detect the HPV positivity and their genotypes. The slide examination results, and demographic data of the patients were retrieved from the Thumbay laboratory, TUH, Ajman, UAE. | |
|  |  | (*b*) Provide in the abstract an informative and balanced summary of what was done and what was found | **2** | A balanced summary and information of the results were provided in the result section of abstract.  “A total of **191 HPV genotypes** were detected in **96 PAP** smear samples collected **(41.92%)** included 47 abnormal cytological and 49 normal epithelial samples. **51 LR** and **140 HR HPV** genotypes were identified in all the 96 positive samples. Highest rate of infection with multiple LR and HR HPV genotypes were detected in women aged 31-40 years **(45%)**. | |
| Introduction | | | |  |  |
| Background/rationale | 2 | Explain the scientific background and rationale for the investigation being reported | **4** | The scientific background and rationale of the current study is given in the Introduction part as mentioned below.  “Some studies reported that the high-risk HPV genotypes are available from the countries with somewhat similar geography and demography to the UAE.  In the UAE, the crude incidence rate of HPV-related cancer is 4.03 per 100,000 people. However, there is a lack of clear data on the prevalence of low and high-risk HPV in the country's general population. Multiple HPV genotypes have been observed occurring in the same individual, as documented [13,14]. This could involve a combination of HR genotypes together or HR and LR genotypes. Identifying the HPV genotype among infected individuals is essential for raising awareness about HR and LR genotypes. Implementing routine screening for at-risk individuals can help reduce the incidence of malignancy. | |
| Objectives | 3 | State specific objectives, including any prespecified hypotheses | **2** | The aim of this study was to determine the genotypic distribution and molecular epidemiology of Human papillomaviruses in PAP smear samples of women in the UAE using peptide nucleic acid-based fluorescence melting curve analysis method. | |
| Methods | | | |  |  |
| Study design | 4 | Present key elements of study design early in the paper | **4, 5** | Determining the genotypic distribution and molecular epidemiology of HPV infection and its genotypes in women with normal and abnormal uterine cervices is critical for effective prevention, early detection, and management of HPV-related diseases, as well as for public health awareness and initiatives”. | |
| Setting | 5 | Describe the setting, locations, and relevant dates, including periods of recruitment, exposure, follow-up, and data collection | **5, 6** | A cross-sectional retrospective study was conducted on the liquid-based cervical cytology samples received from various hospitals of UAE to the Department of Pathology at Thumbay laboratory, Thumbay University Hospital (TUH), Ajman, UAE to diagnose the cervical abnormalities and detect the HPV positivity and their genotypes. In accordance with the IRB approval, the data of the study samples and demographic data of the patients were **retrospectively** obtained from the medical records of Thumbay Laboratory, TUH, starting the period from **January 10, 2024** to **January 10, 2025**. | |
| Participants | 6 | (*a*) *Cohort study*—Give the eligibility criteria, and the sources and methods of selection of participants. Describe methods of follow-up  *Case-control study*—Give the eligibility criteria, and the sources and methods of case ascertainment and control selection. Give the rationale for the choice of cases and controls.  *Cross-sectional study*—Give the eligibility criteria, and the sources and methods of selection of participants | **5, 6** | In this **cross – sectional retrospective studies** no participants were directly recruited. Liquid-based cervical cytology samples collected the women aged 20 – 55 years attending the Gynaecology out-patient department of Thumbay University Hospital and other hospitals of UAE, processed for the routine cytological examination to identify and differentiate morphological changes and detect the HPV positivity and their genotypes. In this **cross sectional -retrospective study** the **patient’s informed consent is waived,** and patient demographic data (Nationality, age) were documented, and other details were kept anonymous. | |
|  |  | (*b*) *Cohort study*—For matched studies, give matching criteria and number of exposed and unexposed  *Case-control study*—For matched studies, give matching criteria and the number of controls per case | **14, 15** | A total of **04 different age groups** were concluded in this study population. **i) 20 -30 years old, ii) 31 – 40 years old, iii) 41 – 50 years old and iv) above 50 years old**. Different age group and ethnicity of the HPV positive patient were given in the result section – “**Age group and Ethnicity of the HPV positive patient”.** | |
| Variables | 7 | Clearly define all outcomes, exposures, predictors, potential confounders, and effect modifiers. Give diagnostic criteria, if applicable | **17, 18** | Out of 229 PAP smear samples, 27.1% samples showed abnormal cytology. Of the abnormal cytology samples, 17.03%, 8.73% were ASCUS and LSIL, 0.87% samples were AGC, and 0.43% was ASC-H each respectively. The abnormal cervical cytology rate has increased nearly 60% in recent years in the UAE when compared with previous studies which was carried out in 2019. The current prevalence of cervical cytological abnormalities was 27.1%. Prevalence of HPV in this study was 41.9% (in abnormal & normal cytology; 20.5% & 21.4%), this was lesser than a previous study (60.6%) which was conducted in 2023 in UAE and this lesser rate has been shown a significant positive awareness of the HPV infection and vaccination among the population. | |
| Data sources/ measurement | 8* | For each variable of interest, give sources of data and details of methods of assessment (measurement). Describe comparability of assessment methods if there is more than one group | **-** | Each variable of interest, sources of data and details of methods of assessment are given in the **S1 additional document file.** | |
| Bias | 9 | Describe any efforts to address potential sources of bias | **-** | In this study, no potential sources of bias were identified | |
| Study size | 10 | Explain how the study size was arrived at | **5** | A cross - sectional retrospective study was conducted on the liquid-based cervical cytology samples received from various hospitals of UAE to the Department of Pathology at Thumbay laboratory, TUH, Ajman, UAE during the period of **January 10, 2024 to January 10, 2025**, to diagnose the cervical abnormalities and detect the HPV positivity and their genotypes. During the study period, A total of 229 cervical smears samples were collected and all the samples’ results were used in this study. | |

| Quantitative variables | 11 | Explain how quantitative variables were handled in the analyses. If applicable, describe which groupings were chosen and why | **8,**  **9** | ASCUS (Atypical squamous cells of undetermined significance), LSIL (low-grade squamous intraepithelial lesions and AGC (Atypical glandular cell), ASC-H (Atypical squamous cells, cannot rule out high grade squamous intraepithelial lesion) and NILM (Negative for intraepithelial malignancy) were detected in the PAP smear samples with different age group of women (**Table 1** – Grading of PAP smear samples collected from various age group of women and detection of HPV positivity in different abnormal and normal cytology samples were done) and Frequency of single multiple low and high-risk HPV genotypes with different age groups of the study population. The inferential statistical analysis was carried out using bivariate analysis (Chi-square test) with the significance level at *p-*value of <0.05 (**Table 3**). |
| --- | --- | --- | --- | --- |
| Statistical methods | 12 | (*a*) Describe all statistical methods, including those used to control for confounding | **8** | In this study, data were analyzed using IBM SPSS for Windows Version 28.0 (IBM CORP, Armonk, NY, USA). Descriptive statistical tests were carried out and the categorical data are presented with frequency and percentage. The inferential statistical analysis was carried out using bivariate analysis (Chi-square test). As shown in Table 3, with the significance level at a p-value of <0.05. |
|  |  | (*b*) Describe any methods used to examine subgroups and interactions | **11, 16, 17** | Number of single, multiple low and high-risk HPV genotypes were identified in 4 different cytology samples (ASCUS, LSIL, AGC, ASC-H) and NILM were identified with different age group of both Arab and Non-Arab ethnicity were analysed and mentioned in the **table no. 2**.  Frequency of single, multiple low and high-risk HPV genotypes with different age group of study population were analysed and their Chi-Square p value were also identified and mentioned in the **table no. 3** in the manuscript. |
|  |  | (*c*) Explain how missing data were addressed | - | No missing data were encountered during the study |
|  |  | (*d*) *Cohort study*—If applicable, explain how loss to follow-up was addressed  *Case-control study*—If applicable, explain how matching of cases and controls was addressed.  *Cross-sectional study*—If applicable, describe analytical methods taking account of sampling strategy | - |  |
|  |  | (*e*) Describe any sensitivity analyses | - | No sensitivity analyses done in this study |
| Results | | | | |
| Participants | 13* | (a) Report numbers of individuals at each stage of study—eg numbers potentially eligible, examined for eligibility, confirmed eligible, included in the study, completing follow-up, and analysed |  | Numbers of individuals at each stage of study are given in the **S2 additional document file.** |
|  |  | (b) Give reasons for non-participation at each stage | - | - |
|  |  | (c) Consider use of a flow diagram |  | The flow diagram has given in the **S3 additional document file.** |
| Descriptive data | 14* | (a) Give characteristics of study participants (eg demographic, clinical, social) and information on exposures and potential confounders | **6** | The demographic data of the patients such as **nationality** and **their age** **group** are used in this study. |
|  |  | (b) Indicate number of participants with missing data for each variable of interest | - | No missing data of the participants were identified in this study |
|  |  | (c) *Cohort study*—Summarise follow-up time (eg, average and total amount) | - | - |
| Outcome data | 15* | *Cohort study*—Report numbers of outcome events or summary measures over time | - | - |
|  |  | *Case-control study—*Report numbers in each exposure category, or summary measures of exposure | - | - |
|  |  | *Cross-sectional study—*Report numbers of outcome events or summary measures | - | Report of number of outcome events has given in the **S4 addition document file.** |
| Main results | 16 | (*a*) Give unadjusted estimates and, if applicable, confounder-adjusted estimates and their precision (eg, 95% confidence interval). Make clear which confounders were adjusted for and why they were included | - | In this study, no adjusted estimates are added. |
|  |  | (*b*) Report category boundaries when continuous variables were categorized | - | - |
|  |  | (*c*) If relevant, consider translating estimates of relative risk into absolute risk for a meaningful time period | - |  |

| Other analyses | 17 | Report other analyses done—e.g., analyses of subgroups and interactions, and sensitivity analyses | **9, 11**  **11**  **16**  **13**  **13**  **17** | After cytological grading of all the PAP smear slides into ASCUS, LSIL, AGC, ASC-H and NILM were grouped according to the patients’ age groups (20-30, 31-40, 41-50 & above 51) and further analysed for the HPV positivity, and it is given in the **table 1** of the submitted manuscript. The patients were further grouped into Arab and non-Arab (**Table 2**).  Number of single, multiple low and high-risk HPV genotypes were further analysed in all the HPV positive ASCUS, LSIL, AGC, ASC-H and NILM of the PAP smear samples were analysed and given in the **table 2**.  Frequency of single, multiple low and high-risk HPV genotypes with different age group of Arab and non-Arab study population were further analysed in all the HPV positive samples and given in the **table 3 & figure 7.**  Number of single and multiple, low and high-risk genotypes detected in different cytology samples is given in the **figure 5**.  Overall frequency of HPV genotypes in all the HPV positive samples including mixed low and high-risk genotypes were further analysed and given in the **figure 6.**  Number of study population with different nationalities and their HPV positivity rate is given in the **figure 8.** |
| --- | --- | --- | --- | --- |
| Discussion | | | | |
| Key results | 18 | Summarise key results with reference to study objectives | **2,**  **3** | In total, 191 HPV genotypes were detected in 96 PAP smear samples (641.92%) included 47 abnormal cytological and 47 NILM samples. 51 LR and 140 HR HPV genotypes were identified in all the 96 positive samples. Highest rate of infection with multiple LR and HR HPV genotypes were detected in women aged 31-40 years (45%). Infection by HR-HPV53, 16, 31, 68, 66, 35 & 45 and the LR-HPV6, 61, 11,44 & 81 were the most common genotypes in the study samples. Co-infection with multiple low and high-risk genotypes is present in 26.2% cases; in that, HPV53 was the most common followed by HPV35, 66, 11, 43, 81, 61 and 6. Non-Arab study population showed higher HPV infection rate than the Arab cohort. Based on molecular genotyping, 21.4% of normal epithelia tested positive infections. This finding highlights the importance of molecular genotyping to emphasize HPV screening triage. |
| Limitations | 19 | Discuss limitations of the study, taking into account sources of potential bias or imprecision. Discuss both direction and magnitude of any potential bias | **20** | The study samples received from various hospitals, clinics and Thumbay hospitals located in and around the northern emirates and other regions of UAE (Sharjah, Ajman, Umm-Al-Quwain, Dubai and Fujairah) and not received the samples from other emirates (Abu Dhabi & Ras-Al-Khaimah). The details of the HPV vaccination status and other comorbidity conditions of the study population were unclear.  A large population-based study across the UAE is needed to determine the most prevalent genotypes and develop new vaccine strategies to reduce the burden of cervical cancer. |
| Interpretation | 20 | Give a cautious overall interpretation of results considering objectives, limitations, multiplicity of analyses, results from similar studies, and other relevant evidence | - | A cautious overall interpretations of the results given in the **S5 additional document file.** |
| Generalisability | 21 | Discuss the generalisability (external validity) of the study results | **19, 20** | The current findings confirm that the UAE has a lower HPV prevalence (42%) compared to a recent study (60.6%) conducted in 2023. According to the study, HRHPV53, 16, 31, 68, 66, 35, 45 and LR6, 61,11, 44 and 81 were the most common HPV infections in the women between 20-55 years old and much less prevalence of HPV 18 was found. A moderate increase in the incidence of HPV 53, 16, 31, 68, 66, 6, 61 & 11 was detected. Co-infection with multiple low and high-risk genotypes was present in 26.2% cases, with HPV 53 being the most common genotype. Based on molecular genotyping, 21.4% of normal epithelia tested positive for HPV infections. It is evident that symptomatic women, even with normal epithelia have been infected with different low and high-risk genotypes. The non-Arab study cohort showed a higher HPV positive rate than the Arab cohort which could be due to a multinational floating population with an unknown HPV screening and vaccination status. Therefore, the present study highlights the importance of molecular genotyping to emphasize HPV screening triage and follow-up with periodic PAP smears for detecting any cytological changes of cervix. |
| Other information | |  | | |
| Funding | 22 | Give the source of funding and the role of the funders for the present study and, if applicable, for the original study on which the present article is based | - | - |

*Give information separately for cases and controls in case-control studies and, if applicable, for exposed and unexposed groups in cohort and cross-sectional studies.

**Note:** An Explanation and Elaboration article discusses each checklist item and gives methodological background and published examples of transparent reporting. The STROBE checklist is best used in conjunction with this article (freely available on the Web sites of PLoS Medicine at http://www.plosmedicine.org/, Annals of Internal Medicine at http://www.annals.org/, and Epidemiology at http://www.epidem.com/). Information on the STROBE Initiative is available at www.strobe-statement.org.
