# Supplementary material for: Genotypic distribution and molecular epidemiology of HPV in women in the UAE using PNA-based RT PCR
Source: PLoS One. 2026 Mar 31;21(3):e0346052. doi: 10.1371/journal.pone.0346052 (PMC13037986; doi:10.1371/journal.pone.0346052)
Supplement: S1 Table — (DOCX) [file pone.0346052.s007.docx]

**Supplementary Table 1.** Baseline cut off (threshold) of melting peak analysis of each fluorescent dye in each HPV tube.

| **Sl.No** | **HPV Mix** | **Fluorescent Dye** | **Baseline cut-off criteria** | |
| --- | --- | --- | --- | --- |
|  |  |  | **CFX96** | **QS5** |
| 1 | HPV Mix #A | FAM | ≥100 | ≥10,000 |
| 2 |  | HEX (or VIC) | ≥50 | ≥5,000 |
| 3 |  | ROX | ≥50 | ≥5,000 |
| 4 |  | Cy5 | ≥150 | ≥15,000 |
| 5 | HPV Mix #B | FAM | ≥100 | ≥10,000 |
| 6 |  | HEX (or VIC) | ≥50 | ≥5,000 |
| 7 |  | ROX | ≥50 | ≥5,000 |
| 8 |  | Cy5 | ≥80 | ≥8,000 |
| 9 | HPV Mix #O | FAM | ≥100 | ≥10,000 |
| 10 |  | HEX (or VIC) | ≥50 | ≥5,000 |
| 11 |  | ROX | ≥70 | ≥7,000 |
| 12 |  | Cy5 | ≥100 | ≥10,000 |
