# Supplementary material for: Genotypic distribution and molecular epidemiology of HPV in women in the UAE using PNA-based RT PCR
Source: PLoS One. 2026 Mar 31;21(3):e0346052. doi: 10.1371/journal.pone.0346052 (PMC13037986; doi:10.1371/journal.pone.0346052)
Supplement: S2 Table — (DOCX) [file pone.0346052.s008.docx]

**Supplementary Table 2.** Criteria for target HPV detection.

| **HPV Mix # A** | | | **HPV Mix # B** | | | **HPV Mix # O** | | |
| --- | --- | --- | --- | --- | --- | --- | --- | --- |
| Type | Fluorescent  Dye | Tm range (ºC) | Type | Fluorescent  Dye | Tm range (ºC) | Type | Fluorescent  Dye | Tm range (ºC) |
| HPV33 | FAM | 45.0 - 49.5 | HPV53 | FAM | 48.0 - 56.0 | HPV32 | FAM | 43.0 - 50.0 |
| HPV11 |  | 55.0 - 60.0 | HPV35 |  | 60.0 - 65.0 | HPV42 |  | 54.0 - 60.0 |
| HPV58 |  | 60.5 - 67.0 | HPV39 | HEX | 47.0 - 51.0 | HPV34 |  | 62.0 - 68.0 |
| HPV16 | HEX | 45.0 - 50.0 | HPV59 |  | 52.0 - 55.0 | HPV61 | HEX | 43.5 - 49.0 |
| HPV73 |  | 61.0 - 65.0 | HPV31 |  | 58.0 - 64.0 | HPV81 |  | 53.0 - 58.5 |
| HPV45 | ROX | 43.0 - 49.0 | HPV68 | ROX | 39.0 - 49.5 | HPV72 |  | 59.0 - 71.0 |
| HPV18 |  | 56.0 - 61.0 | HPV70 |  | 50.0 - 55.0 | HPV40 | ROX | 42.0 - 52.0 |
| HPV69 |  | 66.0 - 70.0 | HPV56 |  | 56.0 - 65.0 | HPV44 |  | 55.0 - 60.5 |
| HPV26 | Cy5 | 42.0 - 46.0 | HPV51 | Cy5 | 43.0 - 47.0 | HPV43 |  | 62.0 - 67.0 |
| HPV6 |  | 50.0 - 55.0 | HPV66 |  | 50.0 - 55.0 | HPV54 | Cy5 | 52.0 - 56.0 |
| HPV52 |  | 59.5 - 64.0 | HPV82 |  | 58.0 - 62.0 | HBB |  | 65.0 - 73.0 |

* Tm values are rounded to second decimal places and applied to the criterion.

* Tm range *"*A - B*"* means *"*A ≤ Tm ≤ B".
