# Supplementary material for: Genotypic distribution and molecular epidemiology of HPV in women in the UAE using PNA-based RT PCR
Source: PLoS One. 2026 Mar 31;21(3):e0346052. doi: 10.1371/journal.pone.0346052 (PMC13037986; doi:10.1371/journal.pone.0346052)
Supplement: S3 Table — (DOCX) [file pone.0346052.s009.docx]

**Supplementary Table 3.** Detection of HPV infection in different cytology grading in various age groups of the study population.

| **Different age**  **Groups**  *n*=229 | **Ar/**  **NAr** | **Different Cytology of PAP smear samples** *n*=229 | | | | | | | | | | |
| --- | --- | --- | --- | --- | --- | --- | --- | --- | --- | --- | --- | --- |
|  |  | **ASCUS**  *n*=39 (17%) | | **LSIL**  *n*=20 (9%) | | **AGC**  *n*=2 (0.9%) | | **ASC-H**  *n*=1 (0.4%) | | | **NILM**  *n*=167 (73%) | |
|  |  | **HPV**  **+ve**  *n*=26 (11%) | **HPV**  **-ve**  *n*=13  (6%) | **HPV**  **+ve**  *n*=18  (8%) | **HPV**  **-ve**  *n*=2  (1%) | **HPV**  **+ve**  *n*=2  (1%) | **HPV**  **-ve**  *n*=0 | **HPV**  **+ve**  *n*=1  (0.5%) | **HPV**  **-ve**  *n*=0 | **HPV**  **+ve**  *n*= 49  (21%) | | **HPV**  **-ve**  *n*=118  (52%) |
| **20-30**  *n*=77 (33.6%) | **Ar**  *n*=40  (17.5%) | 5  (2%) | 10  (4%) | 5  (2%) | 1  (0.5%) | 0 | 0 | 0 | 0 | 7  (3%) | | 12  (5%) |
|  | **NAr**  *n*=37  (16%) | 5  (2%) | 3  (1%) | 4  (2%) | 1  (0.5%) | 0 | 0 | 1  (0.5%) | 0 | 15  (7%) | | 8  (3.5%) |
| **31-40**  *n*=96  (42%) | **Ar**  *n*=44  (19.21%) | 7  (3%) | 0 | 1  (0.4%) | 0 | 1  (0.5%) | 0 | 0 | 0 | 8  (3.5%) | | 27  (12%) |
|  | **NAr**  *n*=52  (23%) | 5  (2%) | 0 | 4  (2%) | 0 | 1  (0.5%) | 0 | 0 | 0 | 13  (6%) | | 29  (13%) |
| **>41**  *n*=56  (24%) | **Ar**  *n*=30  (13%) | 1  (0.4%) | 0 | 1  (0.4%) | 0 | 0 | 0 | 0 | 0 | 3  (1%) | | 25  (11%) |
|  | **NAr**  *n*=26  (11%) | 3  (1.3%) | 0 | 3  (1%) | 0 | 0 | 0 | 0 | 0 | 3  (1%) | | 17  (7%) |

*Ar – Arab, NAr – Non-Arab, ASCUS – Atypical squamous cell undetermined significance, LSIL – Low-grade intraepithelial lesion, AGC- Atypical glandular cell, ASC-H -Atypical squamous cells, cannot rule out high-grade squamous intraepithelial cells, NILM – Negative for intraepithelial malignancy
