# Supplementary material for: Genotypic distribution and molecular epidemiology of HPV in women in the UAE using PNA-based RT PCR
Source: PLoS One. 2026 Mar 31;21(3):e0346052. doi: 10.1371/journal.pone.0346052 (PMC13037986; doi:10.1371/journal.pone.0346052)
Supplement: S4 Table — (DOCX) [file pone.0346052.s010.docx]

**Supplementary Table 4.** Number of single, multiple low and high-risk HPV genotypes in different cytology samples and different age groups of the study population.

| **Age**  **group**  ***n* (%)** | **Ethnicity**  ***n*** | **HPV result +ve/-ve** | **cytological findings**  ***n*** | **HPV +ve infection** *n*=96 | | | | | **Total No. of genotypes** *n*=191 |
| --- | --- | --- | --- | --- | --- | --- | --- | --- | --- |
|  |  |  |  | **Single genotype cases** *n*=46 | | **Mixed genotype cases** *n=50* | | |  |
|  |  |  |  | **Single HR**  *n*=32 | **Single LR**  *n*=14 | **Multiple HR**  *n*=23 | **Mixed LR**  *n*=2 | **Mixed H&LR**  *n*=25 |  |
| **20-30** *n=77*  *(34%)* | **Ar**  *n=40*  *(18%)* | +ve  *n=17 (7%)* | NILM  *n=7 (3%)* | HPV16 (2) HPV45 *n=3* | HPV43 HPV61  *n=2* | HPV16,53 HPV16,66 *n= 2* |  | **-** | 9  *(5%)* |
|  |  |  | LSIL  *n=5 (2%)* | HPV53 *n=1* | **-** | HPV56,66 HPV39,56,66,  68 *n=2* | - | HPV59,11 HPV16,53,59,11,  43,44  *n=2* | 15  *(8%)* |
|  |  |  | ASCUS  *n=5 (2%)* | HPV53  *n=1* | - | HPV18,31,35 HPV16,35,68  HR 16, 35, 53 *n=3* | - | HPV45,52,44 *n=1* | 13  *(7%)* |
|  |  | - ve   *n=23 (10%)* | NILM  *n=12 (5%)* | - | - | - | - | - | - |
|  |  |  | ASCUS  *n=10 (4.5%)* | - | - | - | - | - | - |
|  |  |  | LSIL  *n=1 (0.5%)* | - | - | - | - | - | - |
|  | **NAr**  *n=37*  *(16%)* | +ve *n=25 (11%)* | *NILM*  *n=15*  *(6.6%)* | HPV45 (2) HPV68 (3) HPV18 (2)  *n=7* | HPV61 (2) HPV43 *n=3* | HPV45,51,82 HPV51,31,39 HPV33,53 *n=3* | - | HPV68,43,81  HPV62,44 *n=2* | 23  *(12%)* |
|  |  |  | LSIL  *n=4*  *(2%)* | HPV66  *n=1* | - | HPV58,73  HPV34,35  *n=2* | - | HPV82,61  *n=1* | 7  *(3.7%)* |
|  |  |  | ASCUS  *n=5*  *(2%)* | HPV51 HPV16  *n=2* | - | HPV16,51,58 HR59,73 HPV18,31,53,  58,68  *n=3* | - | - | 12  *(6.3%)* |
|  |  |  | ASC-H  *n= 1 (0.4%)* | HPV68  *n=1* | - | - | - | - | 1 *(0.5%)* |
|  |  | -ve  *n=12*  (5*%*) | NILM  *n=8 (3.5%)* | - | - | - | - | - | - |
|  |  |  | ASCUS  *n=3 (1%)* | - | - | - | - | - | - |
|  |  |  | LSIL  *n=1 (0.4%)* | - | - | - | - | - | - |
| **31 - 40** *n=96*  *(42%)* | **Ar** *n=44*  *(19%)* | +ve *n=17*  *(7%)* | NILM  *n=8*  *(3.5%)* | HPV18 HPV53 *n=2* | HPV6  *n=2* | HPV52,58 *n=1* | - | HPV51,81 HPV53,42  HPV35,6  *n=3* | 12  *(6%)* |
|  |  |  | ASCUS *n=7*  *(3%)* | HPV31  *n=1* | HPV6  *n=2* | HPV33,53  *n=1* | - | HPV31,61 HPV53,61 HPV16,18,31,35, 53,82,43,61,68 *n=3* | 18  *(9%)* |
|  |  |  | LSIL  *n=1 (0.4%)* | HPV53  *n=1* | - | - | - | - | 1 *(0.5%)* |
|  |  |  | AGC  *n=1 (0.4%)* | HPV31  *n=1* | - | - | - | - | 1 *(0.5%)* |
|  |  | **-**ve  *n=27*  *(12%)* | NILM  *n=27 (12%)* | - | - | - | - | - | - |
|  | **NAr** *n=52*  *(23%)* | +ve  *n=23* *(10%)* | NILM  *n=13*  *(5.7%)* | HPV18 HPV70 HPV45  HPV35  *n=4* | HPV42  HPV72 *n=2* | HPV56,66 HPV16,31 *n=2* | - | HPV66,6 HPV16,18,66,6 HPV51,81 (2) HPV31,52,11  *n=5* | 23  *(12.04%)* |
|  |  |  | ASCUS *n=5 (2%)* | HPV58 *n=1* | HPV6 *n=2* | HPV56,66  *n=1* | - | HPV35,45,68,54 *n=1* | 9 *(4.71%)* |
|  |  |  | LSIL *n=4 (2%)* | - | - | - | - | HPV45,59,6 HPV35,52,68, 11,42  HPV53,58,66, 43,61  HPV26,31,35, 45,56,66,11,43  *n=4* | 21  *(11%)* |
|  |  |  | AGC *n=1 (0.4%)* | - | - | - | - | HPV51,81 *n=1* | 2 *(1%)* |
|  |  | -ve  n=29 *(13%)* | NILM  *n=29 (13%)* | - | - | - | - | - | - |
| **≥41**  *n=56*  *(24.4%)* | **Ar** *n=30*  *(13%)* | +ve *n=5*  *(2%)* | ASCUS *n=1(0.4%)* |  |  |  |  | HPV66,6  *n=1* | 2  *(1%)* |
|  |  |  | NILM  *n=3 (1%)* | HPV59 *n=1* | HPV11 *n=1* | HPV31,68 *n=1* | - | - | 4 *(2%)* |
|  |  |  | LSIL  *n=1 (0.4%)* | HPV66 *n=1* | - | - | - | - | 1 *(0.5%)* |
|  |  | -ve *n=25* *(11%)* | NILM  n=25 *(11%)* | - | - | - | - | - | - |
|  | **NAr** *n=26* *(11.4%)* | +ve *n=9*  (4%) | NILM  *n=3 (1%)* | HPV70, 73  *n=1* | - | HPV33,53 *n=1* | - | - | 4 *(2%)* |
|  |  |  | ASCUS  *n=3 (1%)* | HPV59  *n=1* | - | HPV16,31,73 *n=1* | - | HPV53,82,34 *n=1* | 7  *(4%)* |
|  |  |  | LSIL  *n=3*  *(1%)* | HPV16 *n=1* | - | - | HPV42,44 HPV43,61,  81  *n=2* | - | 6  *(3%)* |
|  |  | -ve *n=17 (6%)* | NILM  *n=17 (6%)* | - | - | - | - | - | - |
