# Supplementary material for: Genotypic distribution and molecular epidemiology of HPV in women in the UAE using PNA-based RT PCR
Source: PLoS One. 2026 Mar 31;21(3):e0346052. doi: 10.1371/journal.pone.0346052 (PMC13037986; doi:10.1371/journal.pone.0346052)
Supplement: Attachment — Submitted filename: Response to Reviewers (Rebuttal Letter).docx [file pone.0346052.s012.docx]

**Rebuttal Letter**

**From**

**Dr. NAZEERULLAH RAHAMATHULLAH B.Sc, M.Sc, Ph.D**

Assistant Professor of Microbiology & Immunology

Department of Biomedical Sciences, College of Medicine

Gulf Medical University,

Ajman, United Arab Emirates.

**To**

**THE EDITOR-IN-CHIEF**

PLOS ONE

San Francisco, CA 94111

United States.

**Sub**: Response to Reviewers Manuscript entitled “Genotypic Distribution and Molecular Epidemiology of Human Papillomaviruses in PAP Smear Samples of women in the United Arab Emirates (UAE): Peptide nucleic acid-based fluorescence melting curve analysis study” - [PONE-D-25-39382]

**Dear Sir/Madam,**

Greetings!

On behalf of all the authors, I would like to thank the reviewers and the academic editor for taking the time to thoroughly review our manuscript. As the corresponding author, I have addressed all the queries, suggestions, and corrections raised by the reviewers. Based on their individual comments, I have made the necessary modifications, corrections (additions/deletions), which have been incorporated into the updated manuscript. I have added the author's response for all reviewers and the academic editor’s comments, along with this rebuttal letter. The revised manuscript in the Word format, with and without highlights, has been attached to the PLOS One Journal author’s site.

If anything needs to be added or modified in the updated manuscript, please let us know, and we will do so accordingly.

Thank you for considering our submission.

We look forward to your response.

**Responses to the Reviewers**

**Responses to the Editor's Comments**

1) There is significant textual overlap between the current study and the preceding study (reference 13).

Odeh et al. (2023) Exploring the prevalence of Human Papillomavirus (HPV) genotypes in PAP smear samples of women in northern region of United Arab Emirates (UAE): HPV Direct Flow CHIP system-based pilot study. PLOS ONE 18(9):e0286889. <https://doi.org/10.1371/journal.pone.0286889>

**Answer:** The current study and the study by Odeh et al. were conducted in the same geographical region; however, they differ in time period and study population. Although both studies employed the same study design, the present study includes a larger number of provinces compared to the previous work. Importantly, the current study does not incorporate data from the earlier study population or from referral patients. In the revised manuscript, textual overlap with our previously published 2023 study (Odeh et al.) has been eliminated to ensure originality and clarity.

2) Some paragraphs are identical in both manuscripts. The presentation of several figures and tables appears to be substantially identical as well. Beyond removing the technical overlap, it is also necessary to provide some justification as to why the new (replication) study was needed as per PLOS criteria for publication subheading “Replication Studies”https://journals.plos.org/plosone/s/criteria-for-publication (same goal, same population, same methodology except the last HPV typing assay step, same layout and presentation, only slight details in the conclusion with much of the text copy pasted verbatim).

**Answer**: Replication studies

a) The objectives of the current study align with those of the previous study conducted by Odeh et al.; however, the study populations are distinct and were not referenced in the earlier work. The present study includes a larger sample size (n = 229) comprising participants of diverse nationalities. Individuals with a prior HPV diagnosis, referral cases, and follow-up patients were excluded from the current study to ensure a population of newly screened participants.
b) Cytological screening in the current study was performed using the SurePath liquid-based cytology system, and cytological grading was carried out according to the 2014 Bethesda System. The same approach was applied to all Pap smear samples collected using liquid-based cervical cytology and was consistent with the methodology used in the previous study to diagnose cytological abnormalities. However, HPV genotyping in the current study was conducted using a real-time PCR (RT-PCR) technique, whereas the previous study employed a reverse hybridization method.
c) In the manuscript, Table No. 1 has been shifted to the supplementary file as recommended by the editor. Table no.2 has a similar type of variables to the previous study conducted by us, but with different study populations at a different time period in the same region. Table no 3 has been added in the new format with a statistically significant association between age groups, nationality, and HPV status among participants, and with a strong There was a very strong, statistically significant association between HPV genotypes and HPV status. The above-mentioned tables and statistical analysis are given in the updated manuscripts – **Table 2.** Association of age groups, nationality, and genotypes with HPV status. Line no. 260.
3) P6 L 112-128. Study design/Data collection/Population sections.
i) Are the 229 samples all samples collected in 12 months from multiple hospitals?

**Answer**: In this study, A total of 229 PAP liquid-based cytological samples were collected from hospitals across multiple regions of the UAE, including the provinces of Ajman, Sharjah, Dubai, Umm Al Quwain, and Fujairah, for HPV screening and associated cytological evaluation during the study period from Jan 2024 to Jan 2025. All samples were processed at the Department of Pathology and Microbiology, Thumbay Laboratory, Thumbay University Hospital (TUH), Ajman, UAE.

ii) Information about inclusion and exclusion criteria is missing.

**Answer:** The inclusion and exclusion criteria of the study samples are updated in the manuscript, line no. 133 – 137, 147-149 & 151 - 153.

iii) What were the reasons patients visited hospitals (line 113)? Was this a screening or a referral population?

**Answer:** The study populations who visited the hospitals for primary screening purposes due to the following symptoms: watery discharge, bleeding, pelvic pain, and pain during sexual intercourse. Those study populations are considered, and the patients with a known history of HPV infection or cervical cancer, as well as those previously diagnosed with HPV and undergoing routine follow-up examinations, were excluded from this study.

4) P8 L168-169 the assay information (RealTyperTM HPV 32 Genotyping kit) is not consistent with reference 17 provided for the method (“detecting 40 types”). Assay version should be disclosed in the methods section in case the company makes further changes to the product sold.

**Answer:** The correct reference link for the assay information (RealTyperTM HPV32 Genotyping kit) has been incorporated in the reference section of the revised manuscript. Reference no: 17. Line no. 484

<https://www.hlbpanagene.com/_ENG/html/dh_product/prod_view/86/?cate_no=2>.

5) P8 L147 assay name doesn’t need to be repeated in the same paragraph.

**Answer:** In P8, L147, the assay name has been removed from the sentence, and the updated sentence is mentioned in the manuscript, line no. 167 – 169.

6) P9 results section. The study presents numerous highly overlapping tables (3) and figures (9) that often show high granularity of the data (ie, Table 1 has 80 cells of data, of which only 16 have 5 or more cases). Instead, consider including a supplementary Excel table with detailed information and focus the results section only on critical findings. Also, it is very difficult to find the overall prevalence of individual types, which are later referred to in the text.

**Answer:** As mentioned in the Editor's comments, **Table 1** (Detection of HPV infection in different cytology grading in various age groups of study population) has been moved into the supplementary Table 3. **Table 3** has been updated with chi-square and p-values for the association of age groups, nationality, and genotypes with HPV status of the study population, in the new **Table 2** - Association of age groups, nationality, and genotypes with HPV status. Line no. 260. **Figure 9** has been removed from the updated manuscript.

7) Percentages should be rounded to 1 decimal place in the results text and tables.

**Answer:** All the percentage values are rounded to 1 decimal place in the entire updated manuscript and but in unavoidable situations, some percentage values are still in one-digit format, like 4.6%.

8) P10 Table 1 has a marginally different age grouping than the previous study. Why was the grouping changed? From a statistical point, the >51 category is too small compared to other categories and could have been combined with the 41-50 one. Alternatively, age grouping of previous large-scale reference studies should be used, and the current data compared to those (ie, later mentioned Bruni et al, 2010)

**Answer:** In the present study, the study populations’ age group was originally categorised into 20 – 30 (n=77), 31-40 (n=96), 41-50 (n=48), and ≥51 (n=8). In the 1^st^ age group (20 -30), almost all the study participants are above 21 years old, except 1 participant is 20 years old. Hence, the age group of the study participants is grouped in the above-mentioned pattern. As referred by the editor, **Bruni et al 2010**, in their study, the sample size was 27,343 under the age group of ≤25 years. But in our study, below ≤25 years old study participants was very low (n=5). So, the study has been grouped into 20 -30-year-olds.

As per the editor’s recommendation, the participants are too small under the age group of 51 and above (≥51; n=8), therefore, this age group has been merged with the 41-50 years old study group and updated as ≥41 years old study group (n=56). The updated age group is mentioned in the revised Tables (1, 2 & 3), results, and discussion section of the updated manuscript.

9) P14 L237 the figure 6 should be replaced by a table (or at least the summary information provided as a supplement table)to make it more readable.

**Answer:** Figure 6 has been replaced as **Table 1**. Frequency of high and low-risk genotypes in HPV positive samples, in the manuscript, Line no. 232.

10) P17 L302-306 the p value was calculated on the total number of positive and negative cases. This is not shown on Table 3, where the same p-value details are shown.

**Answer:** Table 3 has been updated to **Table 2,** which includes additional statistical outputs from the chi-square test, including corresponding *p*-values, to assess the association between the dependent variable (HPV status) and the independent variables (age groups, nationality, and genotypes). This revised table is now incorporated into the updated manuscript, and the statistically significant associations between HPV status and the independent variables are clearly reported in the Results section. Line no. 262 – 272.

11) P18 Discussion. Critically, unless the type of population studied (general screening, referral, convenience) is known, any comparisons with previous studies is impossible to appreciate or understand.

**Answer:** The current study, while comparing with the previous study conducted by our team (Odeh et al., Ref. 13) reported several HPV genotypes were reported in combined forms, such as 62/81, 6/43, 31/68, 44/55, and 52/86. These combination results were due to limitations of the reverse hybridization diagnostic method, which did not allow for precise differentiation of individual genotypes. In contrast, the current study accurately identified each genotype independently using an enhanced diagnostic approach – RT-PCR, confirming individual genotypes such as LR81, 6, 43, and 44, as well as HR31, 68, and 52. It is mentioned in the revised manuscript – line no. 356 – 361.

12) The prevalence data are not put in the context of the world in general. For example, Bruni L et al, Cervical Human Papillomavirus Prevalence in 5 Continents: Meta-Analysis of 1 Million Women with Normal Cytological Findings, The Journal of Infectious Diseases, Volume 202, Issue 12, 2010, Pages 1789–1799, <https://doi.org/10.1086/657321>. or a similar encompassing study.

**Answer:** As mentioned by the editor, similar encompassing data of the most prevalent of HPV genotypes has been categorised region-wise: MENA, Asia, and Central Asia, and given in **Table 3** (Comparative frequency of the most prevalent HPV Genotypes among HPV-positive women with ethnicity and nationality). Line no. 276

The most prevalent HPV genotypes of different geographical regions are also discussed in the discussion section and updated in the revised manuscript. Line no. 314 – 324 & 333 - 355.

13) The most commonly found HPV53 was not put into sufficient context in the discussion or conclusion.

**Answer:** The most commonly encountered genotype HR53 has been discussed with previous studies conducted in the UAE and globally with Sanjos et al (2007), Burni et al (2010), Osmani et al 2025 and a study from China (2023). These discussion parts have been updated in the revised manuscript Line no. 319 – 324.

14) The discussion doesn’t sufficiently address the differences between Arab and non-Arab participants and thus doesn’t clearly state whether any differences are notable or if such differentiation is irrelevant for future studies

**Answer:** The above-mentioned comments are considered, and the difference between Arab and non-Arab participants was clearly mentioned and revised in the discussion section. Line no. 333 – 355.

15) Supplementary file S2 doesn’t answer the question of potentially eligible participants. The file completely overlaps the manuscript text and can be removed as uninformative or revised.

**Answer:** The supplementary file S2 has been revised, and the revised file S2 has been submitted along with the updated manuscript.

**Responses to Reviewer-1 comments**

1) The authors tried to highlight the geographic distribution of HPV genotypes among PAP smear samples by using the melted PCR technology. Their finding underscores the importance of molecular testing for HPV genotyping. One comment to highlight is the distinction of the samples as Arab and non-Arab is a crude data i.e. it won't give a casual inference, as the details of these populations is not known, including the epidemiological profile, sexual history etc.

**Answer:** We appreciate the reviewer’s insightful comment. The current study primarily focused on the initial HPV screening of the study population, categorized into Arab and non-Arab ethnic groups. As this was a retrospective, laboratory-based investigation, detailed epidemiological profiles and sexual history information could not be retrieved from the available medical records. This limitation has now been clearly acknowledged and updated in the revised manuscript. Line no. 410 – 421.

We agree that such data are important and will ensure that future studies incorporate comprehensive epidemiological and sexual history information, obtained with proper informed consent from the study participants.

**Response to Reviewer – 2 comments**

2) Methodological details and transparency

i. The methods section is overly technical and lacks a clear sample size justification.

**Answer:** The sample size of the study population has been updated and clearly mentioned in the subheading – “Study populations” under the Methods section of the updated manuscript. Line No: 143 - 158.

ii) Statistical handling of confounding factors (age, ethnicity) and multiple comparisons is insufficiently explained.

**Answer:** The confounding factors, such as Age and ethnicity of the study population, have been updated in tables 2 & 3 of the revised manuscript.

Table 2. Association of age group, nationality, and genotypes with HPV status. Line no. 260

Table 3. Comparative frequency of the most prevalent HPV genotypes among HPV-positive women with ethnicity and nationality. Line no. 276.

3) Data Presentation

i)	Results are extensive but cumbersome, with multiple overlapping tables and inaccessible figures (hyperlinks not functioning).

**Answer:** In accordance with the recommendations, **Tables 1** & **2** have been moved to the supplementary file, and **Figures 6 and 9** have been removed from the revised manuscript. The content originally presented in Figure 6 has now been reformatted and included as **Table 1. “**Frequency of high and low-risk genotypes in HPV positive samples,” in the updated version. Line no. 232.

All required figures have been prepared in TIF format and submitted as separate files, following the PLOS ONE submission standards. Figure captions remain clearly presented within the main manuscript, while the corresponding figures are provided as separate attachments in the updated submission package. Now all the hyperlinks are functioning.

ii) The text often repeats numerical data rather than synthesizing findings.

**Answer:** In response to the comment regarding repeated numerical data, we have revised the manuscript to minimize redundancy. Essential numerical findings are now consolidated and presented clearly in **Tables 1, 2, and 3** within the main text, while additional supporting tables have been moved to the supplementary file. This restructuring ensures better synthesis of the results and improves the overall readability of the revised manuscript.

4) Interpretation and discussion

The discussion section focuses heavily on descriptive comparisons without critically analyzing why prevalence differences occur, what the observed genotype patterns imply, or how they relate to vaccination or screening policies.

**Answer:** In the revised manuscript, we have clarified the demographic context of the United Arab Emirates, where Emirati nationals constitute approximately 15% of the population, while the remaining 85% comprises expatriates of diverse nationalities, predominantly from Asian countries.

Our analysis revealed that certain HPV genotypes—specifically HR53, HR16, and LR61—were more prevalent among the Arab study group originating from MENA-region countries residing in the UAE. In contrast, genotypes HR68, HR66, LR43, and LR81 were more frequently detected among the Asian study group, which included a considerable proportion of participants from Thailand.

We also highlight that several of the predominant genotypes identified in our study (HR53, HR68, HR66, LR61, LR43, LR81, and HR35) are not covered by the currently available 9-valent HPV vaccine (genotypes 6, 11, 16, 18, 31, 33, 45, 52, and 58). This important observation has been emphasized in the revised manuscript.

Based on these findings, we recommend that national stakeholders consider the region-specific genotype distribution when evaluating future HPV vaccine development or adaptation strategies. Furthermore, we suggest strengthening HPV screening policies and vaccination awareness programs not only for Emirati nationals but also for the diverse expatriate population migrating to the UAE for employment. These revisions have been incorporated into the discussion section of the revised manuscript. – Line no.362 – 386.

5) **Public Health Context:**

There is limited discussion of the implications for HPV vaccination programs or national screening guidelines, missing an opportunity to link molecular findings to preventive health outcomes.

**Answer:** The implications for the HPV vaccination program or national screening guidelines are incorporated as mentioned below in the discussion part of the revised manuscript (Line no. 367 – 371 & 379 – 386).

**Detailed Comments to Authors**

1. **Title and Abstract**
   a. Revise the title for brevity and clarity (e.g. “Genotypic Distribution and Molecular Epidemiology of HPV in Women in the UAE Using PNA-based RT-PCR”).

**Answer:** As per the reviewer’s recommendation, the title of the manuscript has been revised as mentioned below

“Genotypic Distribution and Molecular Epidemiology of HPV in Women in the UAE using PNA-based RT PCR” – Title page of the manuscript – Line no 2 - 3.

b. Streamline the abstract by reducing numerical data in the results and adding a brief mention of study type, sample size, and ethical approval.

**Answer:** The above-mentioned suggestions have been incorporated in the abstract – Method & result sections of the revised manuscript.

- Line no. 27 -29 for study type, sample size, and ethical approval
- Line no. 34 – 40 for reducing the numerical data in the result section
c. Revise the conclusion to emphasize implications for HPV screening and vaccination in the UAE.

**Answer:** The conclusion section of the abstract has been revised and emphasizes the implications of HPV screening and vaccination in the UAE. It is mentioned in the revised manuscript (Line no: 41 – 47).

**2. Introduction**

a. Provide a clearer statement of the research gap and study hypothesis—specifically, the lack of UAE-specific HPV genotype data derived from molecular methods.

**Answer:** Data on the prevalence of HR and LR HPV genotypes associated with cervical cancer progression in the UAE remain limited. The emergence of new HPV genotypes not covered by the currently available 9-valent vaccine presents a potential public health concern, as these genotypes may continue to circulate and pose an increased risk if left undetected and untreated. Importantly, many of these emerging genotypes cannot be reliably identified using conventional diagnostic methods. Therefore, comprehensive molecular surveillance using real-time PCR is essential to ensure accurate detection of circulating HPV genotypes. This statement is mentioned in the introduction section of the revised manuscript. (Line no.91 – 103)

b. Expand on the justification for using PNA-based fluorescence melting curve analysis, highlighting its advantages over conventional genotyping (e.g., increased sensitivity, multiplexing capability).

**Answer:** The PNA-based fluorescence melting curve analysis utilizes peptide nucleic acid probes that possess high target specificity, enabling precise hybridization to their complementary sequences. This high specificity allows for the accurate detection of individual targets and supports multiplex analysis, making it possible to identify multiple HPV genotypes within a single reaction. It is mentioned in the introduction part of the updated manuscript. (Line no. 96– 103)

c. Ensure citations are correctly formatted and up-to-date, replacing local file paths with proper references.

**Answer:** All the citations are correctly formatted and updated, and the local file paths are properly tagged with references. All the updated citations are given in the reference section of the revised manuscript. Page no. 21, line no - 435 – 597.

**3. Methods**

a) Condense detailed descriptions of DNA extraction and RT-PCR protocols; move extended procedural steps to supplementary files.

**Answer:** As mentioned by the reviewer, the above suggestions are done in the methodology section. The protocol for DNA extraction and RT-PCR has been condensed, and it is given in the method section. (DNA extraction: Line no. 170 – 179 & RT-PCR protocol: Line no. 180 – 190)

b) Include a sample size rationale or post-hoc power discussion to justify the study’s scale.

Answer: the sample size rationale has been added in the updated manuscript – Method section under the subheading “ Data collection & Study population “ – Line no. 132 – 138 & 143 - 153.

c) Describe how unavailable data and potential confounders (age, ethnicity) were managed.

**Answer:** The above-mentioned unavailable data and potential confounders were managed in this study and mentioned in the revised manuscript – Method section in the subheading “Data Collection.” Line no: 133- 142.

d) Present 95% confidence intervals for proportions to enhance analytical robustness.

**Answer:** In the method section -subheading – “Statistical Analysis” – It is mentioned that Binary logistic regression was conducted to determine the predictors of positive HPV status, including age groups and nationalities. The results are summarized in revised manuscript in table no. 4. Line no: 200 -202 & 281 - 289.

e) Expand the statistical analysis section: specify tests used, significance thresholds, and handling of multiple comparisons.

Answer: In the method section -subheading – “Statistical Analysis” – It is mentioned that a Chi-square test was conducted to determine the association between the dependent variable, HPV status, and the independent variables: age group, nationality, and genotypes. the significance level was set at a p-value of <0.05. The above-mentioned statistical analysis has been given in **Table 2** of the revised manuscript. Line no: 198 – 200, 260 - 272.

4. **Results**
   a) Simplify presentation by focusing on summary statistics and moving highly detailed breakdowns (such as genotype stratifications) to supplementary tables.

**Answer:** As recommended by the Editor and Reviewers, **Tables no 1 & 2** have been moved to supplementary tables (**S. Table 3 & 4**), and the results have been presented in summary format. The above-mentioned updates are given in the revised manuscripts. Line no: 204- 209, 217 – 227, 234 – 256 & 262 – 275, 284 -289.

b) Interpret key findings (e.g., significance of p-values, age distribution trends) directly in text.

**Answer:** The above-mentioned comments are incorporated in the updated manuscript, Line no: 262 – 272, 284- 289.

c) Include confidence intervals alongside prevalence estimates to communicate statistical certainty.

**Answer:** The confidence intervals are mentioned in **Table 4**. “Predictors of Positive HPV status,” and the descriptions are given in the revised manuscript. Line no: 284 – 289.

d) Highlight main findings succinctly—avoid repeating numerical results from tables verbatim

**Answer:** The main findings are highlighted in the result section under the various subheadings, and the repeated numerical results are verbatim in the tables. As suggested by the editor, **Tables 1 and 2** are moved to supplementary tables. It is given in the updated manuscript from Line no. 205 – 289.

**5.	Discussion**

- Improve the discussion by including:

a. Determinants of prevalence variation within the UAE and in comparison to regional/ international studies.

**Answer**: The prevalence variation within the UAE and compared to the MENA region and international studies as well. These details are mentioned in the updated manuscript discussion section from lines no 297 – 355.

b. Epidemiological interpretation of genotype distribution, particularly the predominance of HPV53.

**Answer:** The epidemiological interpretation of genotype distribution, particularly the predominance of HPV53, was discussed in the discussion section of the manuscript from line no. 314 – 324.

c. Public health implications, including relevance for HPV vaccine coverage and screening triage.

**Answer:** The above-mentioned points of HPV vaccine coverage and screening triage have been discussed in the discussion section of the revised manuscript. Line no. 367 – 386.

d. Study limitations and strengths, with attention to retrospective design and sampling bias

**Answer:** In the limitation of the study section, the above-mentioned details are incorporated in the updated manuscript lines no. 409 – 421.

e. Discuss the biological and vaccine policy relevance of HPV53, which is not covered in current vaccine formulations.

**Answer:** The above-mentioned vaccine policy related to HPV53 has been discussed in the discussion section of the updated manuscript. Line no. 372 – 386.

f. Expand on differences observed between Arab and non-Arab populations, linking these to socio-behavioral or healthcare access factors.

**Answer:** The socio-behavioral or healthcare access factors for the Arab and non-Arab populations have been mentioned in the discussion section of the revised manuscript, line no. 374 – 378.

g. Avoid reiterating numerical data already presented in the Results section.

**Answer:** In the revised manuscript, repetition of numerical data is avoided in the discussion section, which is already presented in the results section. Line no. 291 – 386.

**6. Conclusion**

i) Rewrite the conclusion to be concise and forward-looking

**Answer:** The conclusion of the present study has been revised and incorporated into the updated manuscript. Line no. 387 – 407.

ii) Focus on how this study contributes to the understanding of HPV epidemiology and informs policy and prevention efforts.

**Answer:** The above-mentioned suggestions, already been incorporated into the conclusion section of the revised manuscript. Line no. 399 – 407.

iii) Avoid listing prevalence percentages; instead, summarize key implications and suggest future directions such as:

(a) A UAE-wide HPV surveillance initiative.

(b) Expansion of vaccine formulations to include regionally prevalent genotypes (e.g., HPV53).

(c) Integration of molecular genotyping into national cervical screening programs.

**Answer:** The above-mentioned suggestions are incorporated and updated in the revised manuscript. Line no. 399 – 407.
